# Barriers to Healthcare Access for Homeless Women: Perspectives of Social Intervention Professionals

**DOI:** 10.3390/ijerph22121872

**Published:** 2025-12-16

**Authors:** María Virginia Matulič Domandzič, José Manuel Díaz González, Núria Fustier García, Eliana González Gómez

**Affiliations:** 1 Department of Social Work and Social Services, University of Barcelona, 08007 Barcelona, Spain; 2 Department of Communication Sciences and Social Work, University of La Laguna, 38200 San Cristóbal de La Laguna, Spain; 3 Department of Pedagogy, University of Girona, 17004 Girona, Spain; 4 Canary Islands Healthcare System, 38001 Santa Cruz de Tenerife, Spain

**Keywords:** women experiencing homelessness, social exclusion, gender-based violence, health barriers, professional care

## Abstract

**Highlights:**

**Public health relevance—How does this work relate to a public health issue?**

- Homeless women face compounded health vulnerabilities due to gender-based violence, chronic illness, and mental health conditions that remain largely unaddressed.
- Barriers in access to healthcare deepen health inequities for a population already experiencing extreme forms of social exclusion.

**Public health significance—Why is this work of significance to public health?**

- This study identifies structural, institutional, and attitudinal barriers that limit homeless women’s access to essential healthcare services, highlighting gaps in current public health systems.
- Findings emphasize the need for gender-sensitive, trauma-informed frameworks to ensure equitable healthcare provision for this marginalized group.

**Public health implications—What are the key implications or messages for practitioners, policy makers and/or researchers in public health?**

- Improving access to healthcare requires streamlined administrative procedures, enhanced intersectoral coordination, and training of healthcare professionals to reduce stigma and discriminatory practices.
- Public health policies must integrate specialized services, preventive programs, and gender-informed approaches to effectively safeguard homeless women’s right to health.

**Abstract:**

(1) Background: Female homelessness is one of the most invisible forms of social exclusion, aggravated by structural and gender factors and by experiences of violence. This research analyzes the multifaceted barriers hindering women experiencing homelessness from accessing healthcare services, from the perspective of social intervention professionals. (2) Methods: A qualitative study was conducted using three focus groups with 21 professionals from Santa Cruz de Tenerife, Lleida and Barcelona. An interpretative phenomenological approach guided data collection and analysis, and transcripts were examined through thematic analysis to identify common patterns in professionals’ meaning-making regarding healthcare barriers. (3) Results: Gender-based violence cuts across the life trajectories of most women experiencing homelessness, hindering their access to healthcare services. Barriers identified include lack of documentation, stigma and discriminatory treatment, limited access to specialized services, the absence of a gender perspective in healthcare, and a lack of coordination between social and health services. In addition, the study highlights the lack of preventive programs and health education tailored to this population. (4) Conclusions: It is essential to adopt a comprehensive, intersectional and gender-sensitive approach to safeguard the right to health for these women. Measures such as training for healthcare personnel, simplifying bureaucratic procedures, creating specialized resources, and improving inter-institutional coordination are proposed.

## 1. Introduction

Female homelessness is one of the most invisible forms of social and residential exclusion in Europe and other regions of the world. Unlike men, who tend to represent a higher percentage of the homeless population, women experiencing homelessness face specific trajectories marked by structural, social and gender factors that exacerbate their vulnerability [1]. This phenomenon is not limited to the absence of stable housing, but is also affected by discrimination, previous violence, and barriers to accessing essential resources such as care within the healthcare system [2]. In this vein, Rodríguez Moreno et al. [3] point to gender differences in stressful events, with sexual violence in childhood and abuse by a partner being significantly higher among women.

Despite the increase in interest in homelessness in recent decades, an androcentric view continues to prevail, rendering the situation of women in this condition invisible [4]. Women experiencing residential exclusion often experience “hidden” homelessness [5,6,7], in which they resort to informal networks for temporary shelter to avoid life on the streets, with a predominance of situations categorized as unsafe or inadequate housing [8], which makes it difficult to recognize them in official statistics and public policy designs [1,9,10,11]. As Peña [12] points out, women experiencing residential exclusion rely more than men on informal support networks, staying with family members, friends or even resorting to unwanted sexual relationships. This is largely due to the dangers of living on the streets, such as abuse, street harassment, exploitation in prostitution and other sexual assaults [9,13,14,15]. This situation is quite common among women who suffer gender-based violence. They fear being left with no alternative, especially when they have children to care for, and this forces them to remain in unsafe housing, suffering threats from their aggressors [16].

In addition, they face a higher risk of gender-based violence, abuse, and exploitation, which reinforces their vulnerability and hinders their access to health services [17]. Pleace [18] argues that European statistics on homelessness significantly underestimate the prevalence of female homelessness due to the definitions and methodologies used, although it has been shown that gender-based violence is a trigger for homelessness and that women often use various strategies to escape violent contexts and relationships, which can increase their vulnerability [4,10].

Recent studies have documented how gender-based violence and homelessness are deeply interrelated [1,12,19], highlighting the “triple invisibility” faced by women experiencing homelessness, as women, homeless people and victims of gender-based violence, which further exacerbates their vulnerability [20]. Women experiencing homelessness have not only suffered violence prior to their current situation but continue to be exposed to abuse and exploitation on the streets, in shelters and other places of refuge [3]. Matulič-Domandzič et al. [21] point out that violence runs through the life trajectories of these women, becoming a direct trigger for homelessness. The invisibility of these experiences within social protection systems perpetuates their exclusion and hinders the implementation of effective intervention strategies [22,23].

Recent studies such as that by Botija et al. [24] in Valencia show that people who sleep on the streets have a poorer quality of life and that more than a quarter of the sample do not have a health card, which further hinders their access to health services. Numerous studies show that women experiencing homelessness are at higher risk of premature death [25,26]. One of the main challenges faced by women in accessing the healthcare system is the intersection between structural and attitudinal barriers. The former includes a lack of official documentation, no fixed address and the fragmentation of health and social services [27]. Bureaucracy in health systems makes it difficult for these women to access continuous care, exacerbating their physical and mental health problems. Attitudinal barriers include stigma, discrimination, and the lack of a gender perspective in healthcare. Studies have shown that healthcare personnel may harbor prejudices about women experiencing homelessness, treating them with mistrust, or even dismissing their medical needs [28,29].

Mental health is one of the most affected dimensions in this population; some research cited by Montgomery et al. [26] reports that homeless men are more likely to suffer from substance abuse disorders, while women are more likely to suffer from mental health problems [30,31]. The violence and systematic victimization to which many women experiencing homelessness are exposed increases their risk of developing disorders such as depression, anxiety, or post-traumatic stress disorder [32]. However, mental health services are not always equipped to meet their specific needs, and intervention is often limited to prescribing medication without adequate therapeutic follow-up [17]. Recent research highlights the importance of an intersectional, trauma-informed approach to addressing the mental health of these women [33].

In addition to mental health, the physical health of women experiencing homelessness is also severely affected. Lack of regular access to preventive medical and primary care services contributes to a higher incidence of chronic diseases, infections, and untreated gynecological problems [19,34]. Many women in this situation have difficulty accessing adequate treatment due to lack of health insurance, constant mobility, and ignorance of their health rights [33]. Furthermore, the gender-based violence and sexual exploitation to which they are exposed to contribute to a significant deterioration in their health, increasing their vulnerability to sexually transmitted diseases and unplanned pregnancies without adequate medical follow-up [3].

These women’s right to health is limited by the absence of public policies that address their specific needs from a gender and human rights perspective. The invisibility of their issues in the design of health strategies perpetuates their exclusion from the health system and restricts their ability to access dignified and quality care [17,35,36]. The implementation of health programs tailored to their circumstances, including mobile medical units, access to gynecological services and specialized mental health programs, is key to guaranteeing the exercise of their fundamental rights [33]. Given this reality, Alonso Pardo et al. [16] highlight the importance of creating gender-specific social resources to prevent sexual assault and harassment, thus ensuring safe and supportive spaces for women experiencing homelessness.

From the perspective of social intervention professionals, it is essential that the issue of female homelessness in the healthcare system be addressed from a comprehensive and interdisciplinary approach. Coordination between health services, social work and community support organizations can help to improve access to and quality of healthcare for these women. In addition, intervention strategies need to incorporate a gender perspective to ensure that the specific characteristics of these women are recognized and addressed effectively [21,37].

Studies such as that by Botija et al. [24] show that lack of access to healthcare has a direct impact on the quality of life of women experiencing homelessness, who have a higher prevalence of chronic diseases, disabilities, and a high burden of mental illness. Guillén et al. [38] highlights how discrimination and stigma exacerbate these conditions, further limiting their chances of receiving adequate care. Recent research indicates that homelessness and exposure to extreme street conditions negatively affect the life expectancy of these women, reinforcing the need for specific intervention strategies [26,34].

Given this reality and the evidence in the literature, this research aims to analyze the barriers experienced by women experiencing homelessness in categories 1, 2 and 4 of the ETHOS classification [39] in accessing healthcare from the perspective of social intervention professionals, identifying challenges and proposing strategies to improve their access and quality of care.

## 2. Materials and Methods

### 2.1. Design

This study followed a qualitative, exploratory and descriptive design. An interpretative phenomenological approach (IPA) [40] is adopted, which allows us to understand how these women experience and make sense of their interaction with the healthcare system from the perspective of the social professionals who accompany them in their processes towards social integration. For data collection, we opted for focus groups, as they encourage the exchange of experiences and the collective construction of knowledge. This methodology allows us to identify common patterns and differences in the participants’ experiences, as well as to understand how institutional and attitudinal barriers influence their access to health services [41]. Although Interpretative Phenomenological Analysis (IPA) is most commonly applied through individual interviews, its use in focus groups is considered appropriate when the purpose is to explore shared meaning-making within a professional community [42]. In this study, focus groups enabled participants to collectively reflect on their interpretative frameworks regarding the barriers faced by homeless women in the healthcare system. To mitigate potential limitations associated with group dynamics, such as reduced depth of individual accounts or influence among participants, the moderators encouraged balanced participation, probed individual viewpoints, and validated divergent perspectives. These strategies ensured alignment with IPA principles by maintaining an emphasis on how each participant made sense of their professional experiences while also capturing the collective interpretative processes that emerge in practice.

### 2.2. Sample

The study was conducted in three locations: Santa Cruz de Tenerife, Lleida, and Barcelona. In each location, a focus group was carried out with social intervention professionals working directly with women experiencing homelessness. A total of 21 people participated (6 in Tenerife, 7 in Lleida and 8 in Barcelona) and the sessions lasted an average of 1 h and 41 min.

Participants were selected through purposive sampling, prioritizing professionals with at least one year’s experience in working with women experiencing homelessness. Efforts were made to ensure diversity in the profiles, including social workers, social educators, psychologists, and social integrators, among others. The sessions were held in May 2024 and were moderated by researchers with expertise in homelessness, gender studies, and qualitative methodology, ensuring methodological rigor and thematic depth.

### 2.3. Instrument

For data collection, a semi-structured discussion guide was specifically developed for this research. The guide consisted of eight core open-ended questions and several optional prompts designed to facilitate deeper exploration of participants’ experiences. Its development followed a deductive process, grounded in key theoretical and empirical contributions on homelessness, gender-based violence and healthcare access [2,8,14,16,18,22]. These frameworks informed the selection of dimensions such as structural barriers, stigma and discrimination in healthcare settings, coordination challenges between services, and the health impacts of gender-based violence.

The initial version of the guide was reviewed by four researchers with expertise in qualitative methods, homelessness and gender studies, who suggested adjustments to question order, clarity and conceptual alignment. After incorporating this feedback, the guide was pilot tested with two external professionals to the study population, which allowed refinement of wording and the inclusion of additional probes to deepen reflection. The final guide ensured comprehensive exploration of the barriers experienced by homeless women, with particular attention to the intersection between gender-based violence and healthcare access.

The guiding questions addressed dimensions such as administrative and documentation obstacles, perceptions of treatment by healthcare personnel, difficulties detecting and responding to gender-based violence, and challenges related to inter-institutional coordination. Examples of guiding questions include: Do you think women experiencing homelessness face greater barriers to accessing health services compared to other vulnerable groups? What kind of institutional barriers have you identified in these women’s access to health systems? Can you provide specific examples of cases in which women have experienced rejection or lack of support in this system? Do you think there are gender stereotypes that influence the perception and treatment these women receive from health professionals? What changes or measures do you consider necessary to improve access to health services for women experiencing homelessness?

### 2.4. Procedure

The data collection process was carried out in several stages. First, a review of the existing literature was conducted, and the semi-structured discussion guide was validated through a pilot test. Participants were then contacted through professional networks and organizations working with women experiencing homelessness.

Focus groups were held in meeting rooms previously arranged with collaborating entities, ensuring privacy and a safe environment. Before each session, researchers explained the purpose of the study, addressed questions, and obtained written informed consent. All sessions were audio-recorded and transcribed verbatim in accordance with ethical standards for qualitative research.

To ensure the rigor of the qualitative process, the study followed Lincoln and Guba’s trustworthiness criteria [43]. Credibility was strengthened through analyst triangulation: two researchers independently coded the transcripts and subsequently compared and discussed their interpretations to reach consensus. Probing questions used during the focus groups and the incorporation of detailed field notes contributed additional contextual information that enhanced interpretative depth. Dependability was ensured by maintaining an audit trail documenting analytical decisions, coding iterations and reflexive notes throughout the research process. Confirmability was supported through ongoing reflexive discussions within the research team to ensure that interpretations remained grounded in participants’ accounts rather than researchers’ assumptions. Finally, transferability was facilitated by providing rich descriptions of the study settings, participant characteristics and contextual factors, enabling readers to assess the applicability of the findings to other contexts.

### 2.5. Data Analysis

Data analysis followed an iterative and inductive process consistent with Interpretative Phenomenological Analysis (IPA) and thematic analysis. Two researchers independently conducted line-by-line open coding of the transcripts, identifying meaningful units and developing preliminary codes. Through constant comparison, these codes were refined, grouped and developed into emerging themes.

The team engaged in axial coding to explore relationships between categories and to iteratively construct higher-order conceptual groupings. Throughout the process, researchers used memo writing, reflexive dialogue and repeated returns to the transcripts to ensure analytic depth. Discrepancies were discussed and resolved by consensus. This rigorous and cyclical process facilitated the development of a coherent thematic structure grounded in participants’ accounts.

### 2.6. Ethical Considerations

The necessary measures were taken to ensure the confidentiality of the participants. Participants were informed of the purpose of the study and provided written informed consent prior to the sessions, with the guarantee that participation was voluntary and that they could withdraw at any time without repercussions. The data in the transcripts was anonymized and the information securely stored in a protected environment with access restricted to the research team only. Information was managed in accordance with current data protection regulations in the European Union, specifically the General Data Protection Regulation (GDPR, 2016/679). The study was conducted in accordance with the principles of the Declaration of Helsinki (1975, revised 2013). Ethical approval was obtained from the Comité de Ética de la Investigación y Bienestar Animal de la Universidad de La Laguna, under the registration number CEIBA2025-3686.

## 3. Results

**Figure 1:**
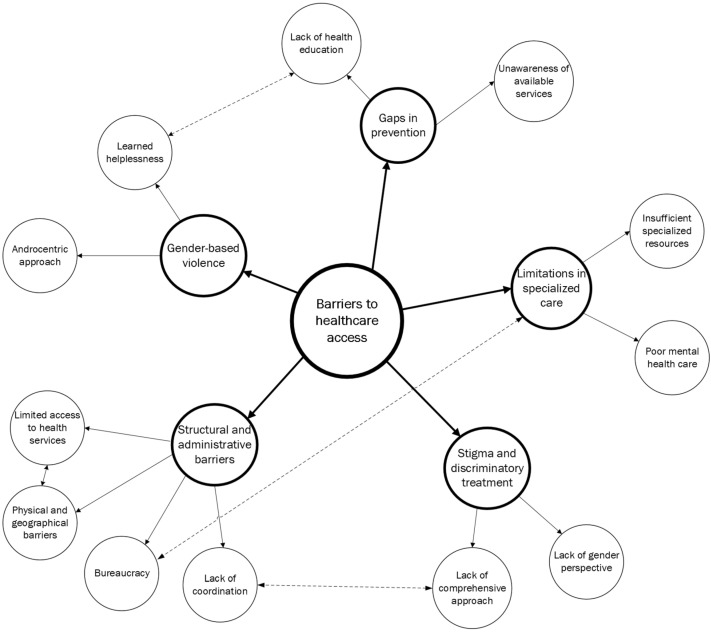
Concept/Thematic map of categories for analysing barriers to accessing the health system for homeless women. Note: Following the optimal concept-map model proposed by Novak & Gowin [44], an additional visual convention (a dashed line) is introduced to indicate a weaker level of linkage between categories.

The thematic analysis of the discussion groups resulted in the identification of five overarching categories that capture the main barriers experienced by women facing homelessness in their access to healthcare: (1) gender-based violence and trauma as structural determinants of health (Section 3.1 and Section 3.7); (2) administrative, legal and bureaucratic barriers to healthcare access (Section 3.2 and Section 3.6); (3) stigma, discriminatory treatment and institutional violence in healthcare settings (Section 3.3); (4) insufficient gender-sensitive, comprehensive and specialized care (Section 3.4, Section 3.5 and part of Section 3.7); and (5) structural barriers to continuity of care, prevention and health promotion (Section 3.8 and Section 3.9). To enhance analytical clarity and illustrate the relationships between these categories, Figure 1 presents a conceptual map summarizing the main barriers and their interconnected subthemes. The following sections present each category in detail, drawing on the accounts of participating professionals.

### 3.1. Gender-Based Violence as a Cross-Cutting Factor in Women’s Lives

The life trajectories of many women experiencing homelessness are marked by different forms of gender-based violence, which may have occurred both before they became homeless and during their process of residential exclusion. A professional from the Tenerife group describes how violence is at the root of many situations of homelessness: “I would dare to say that 100% of the women they have assisted on the streets have been victims of gender-based violence, either currently or at some point in their lives” (Professional 1, Tenerife, May 2024). Professionals point out that many women do not verbalize or report the violence they have suffered out of fear, shame, or mistrust of institutions. One of the participants comments: “A woman who is experiencing violence… does not feel that she can report it and that it is an option for her” (Professional 4, Barcelona, May 2024).

Social resources are often designed from the perspective of men’s needs and, as one participant points out, “intervention methodologies have an androcentric approach” (Professional 3, Tenerife, May 2024). Furthermore, they do not consider the specific nature of gender-based violence and do not have adequate mechanisms to assist these women. One participant commented on this:

For me, it would also be institutional violence, especially the lack of adapted resources, because we don’t only deal with women who are homeless or victims of violence, for example, but there may also be serious problems with substance abuse and mental health, and there are no adapted resources or professionals who have all the tools needed to provide optimal support (Professional 3, Lleida, May 2024).

### 3.2. Limited Access to Health Services

Women experiencing homelessness face administrative and structural barriers that hinder their access to health services, negatively impacting their overall well-being. One of the main barriers is the lack of official documentation, such as a health card, which is a prerequisite for accessing primary care. As one participant pointed out: “Many times they don’t have health card because they are not registered. If you are not in the system, you simply do not exist for primary care” (Professional 2, Barcelona, May 2024).

The absence of a fixed address also represents a considerable obstacle. To register with the primary care system, it is necessary to provide proof of registration with the local authorities. However, even though Article 15.1 of Law 7/1985, Regulating Local Government [45], establishes the duty to register as a resident in Spanish territory and, therefore, a right of citizenship, in practice many homeless people see this right violated. It becomes impossible for women living on the streets or in temporary accommodation to register without first overcoming institutional barriers. This situation prevents them from accessing regular medical check-ups and disease prevention programs. As a result, their health problems worsen, and they are more likely to develop untreated chronic diseases.

### 3.3. Stigma and Discriminatory Treatment

Professionals pointed out that women experiencing homelessness are often stigmatized by healthcare staff, which leads to negative experiences and a deep mistrust of health services. This stigma manifests itself in many ways, including mistrust, dehumanizing treatment, and a condescending attitude towards their situation. As one participant explained: “They look at them as if they are to blame for their situation. There are comments like ‘here again?’. That kind of attitude discourages them from seeking help” (Professional 3, Tenerife, May 2024).

Stigma can manifest itself in derogatory comments about their appearance or hygiene, a lack of empathy towards their health problems, or the assumption that they are looking for drugs or trying to obtain undue benefits. This discriminatory treatment not only makes them feel ashamed and humiliated but also has a direct impact on their health. Women who have experienced stigma in health services are more likely to delay seeking medical care, interrupt their treatment, and avoid contact with the health system in the future. Along the same lines, another professional states: “Many of them tell us that they prefer not to go to the hospital because they feel that they are judged rather than cared for” (Professional 2, Lleida, May 2024).

### 3.4. Lack of a Comprehensive Approach and Gender Perspective

The health system often prioritizes solving immediate medical problems without considering the complexity of the situation of women experiencing homelessness. This translates into insufficient care for their sexual and reproductive health, as well as a lack of specific protocols for caring for women who have experienced gender-based violence. As one professional points out: “Women experiencing homelessness have different needs than men, but the system continues to treat them as if they were the same” (Professional 4, Lleida, May 2024).

A comprehensive approach involves addressing the health needs of these women from a holistic perspective, considering not only their physical problems but also their mental health needs, history of violence and social circumstances. In relation to this issue, one professional explains: “In health centers, no one asks them about their experiences of violence, and this directly affects their physical and mental well-being” (Professional 3, Barcelona, May 2024).

### 3.5. Insufficient Specialized Resources

The lack of resources specifically dedicated to homeless people with a gender perspective significantly limits the care options available to women. This includes the absence of multidisciplinary teams and the shortage of specialized mental health and addiction, two critical areas for addressing the complex needs of these women. In this regard, two professionals argue: “When they need help with addiction or mental health, there is nowhere they can go without waiting months” (Professional 2, Tenerife, May 2024) and “there is no specialized center for women experiencing homelessness with mental health problems in the entire province. That is a huge shortcoming” (Professional 1, Lleida, May 2024).

### 3.6. Bureaucracy and Lack of Coordination

The fragmentation between health and social services creates additional difficulties that prevent women experiencing homelessness from accessing the care they need. These women face complex bureaucratic processes that are not designed for their reality, leaving them outside the system and exposing them to a maze of incomprehensible procedures and requirements. Furthermore, the lack of communication between different sectors (health, social services, housing, etc.) prevents a comprehensive approach to their needs, perpetuating their exclusion and hindering their recovery. One participant reinforces this idea: “They are often referred from one service to another, but there is no coordination. They end up abandoning the process because they feel lost” (Professional 5, Barcelona, May 2024).

An example of this excessive bureaucracy is the difficulty in obtaining the necessary documentation to access medical care. Women experiencing homelessness often lack identity documents, health cards, or registration certificates, and obtaining these documents can be a long and tedious process for them, requiring multiple visits to different offices and the submission of numerous forms. One of the professionals commented: “Bureaucracy is not designed for people who are excluded. It is as if they were condemned to not have access to anything” (Professional 1, Lleida, May 2024).

This bureaucracy and lack of coordination have negative consequences for the health and well-being of these women. Loss of continuity in medical care, duplication of efforts and worsening health problems are just some of the consequences of this situation. As a result, women experiencing homelessness do not receive comprehensive, timely and quality care, which reinforces the urgency of implementing strategies to ensure coordination between different services and simplify bureaucratic processes.

### 3.7. Poor Mental Health Care

Mental health is one of the most neglected areas in the care of women experiencing homelessness. Although many of them have experienced serious trauma, such as gender-based violence, sexual abuse and experiences on the streets, access to mental health services is restricted by long waiting lists, lack of resources and poor training in trauma among healthcare teams. One of the participants sums it up as follows: “Mental health is the big issue. These women are carrying trauma that no one addresses, and that makes it difficult for them to move forward” (Professional 3, Lleida, May 2024).

Trauma and violence have a devastating impact on the mental health of women experiencing homelessness. Many of them suffer from depression, anxiety, post-traumatic stress disorder, and other mental health conditions. These mental health problems can hinder their ability to find housing, employment, and stable relationships, perpetuating their homelessness. Despite this, the focus is often on pharmacological treatments. On this issue, one professional comments: “They are prescribed medication, but there is no follow-up. It is a band-aid, not a solution” (Professional 2, Barcelona, May 2024).

### 3.8. Physical and Geographical Barriers

Professionals highlighted how the location of health centers and opening hours often do not reflect the reality of women experiencing homelessness, making it difficult for them to access medical care. The lack of adequate transport also complicates accessibility. In relation to this issue, one professional stated: “The centers are far away, and they have no way of getting there. Even if they want to go, sometimes they cannot afford public transportation” (Professional 5, Tenerife, May 2024). Similarly, another professional said: “Many services operate at times that do not fit in with their daily routine. It is as if the system were designed to exclude them” (Professional 2, Lleida, May 2024).

### 3.9. Lack of Health Education and Prevention

Women experiencing homelessness are often unaware of the services available to them and their health rights, which limits their ability to access the medical care they need. One professional reinforces this idea with the following argument: “They don’t know that they are entitled to emergency healthcare, even if they don’t have documentation. There is a lot of information missing in this regard” (Professional 4, Barcelona, May 2024). In addition, there are not enough preventive programs specifically targeting them, which increases their vulnerability to disease. One professional comment: “Prevention does not exist for them. No one seeks them out to offer them check-ups or information” (Professional 1, Tenerife, May 2024).

## 4. Discussion

The findings of this study not only confirm the existence of significant barriers faced by women experiencing homelessness in accessing health services but also deepen our understanding of the interpersonal and attitudinal dynamics that perpetuate their exclusion from healthcare. These barriers, identified through the experiences and observations of social workers, include structural and administrative obstacles (such as lack of documentation and fragmentation of services), as well as stigma and discriminatory attitudes on the part of healthcare personnel. They reflect not only the structural and administrative obstacles previously documented in the literature [27,33], but also the persistence of stigma and discriminatory attitudes in the care they receive from the healthcare system [38].

In line with previous studies [4,17], the research shows that female homelessness intersects with gender factors such as gender-based violence, sexual discrimination and unpaid care responsibilities [19,42], which exacerbate these women’s vulnerability and limit their effective access to healthcare services. As pointed out by Alcántara and Arredondo [9] and Bretherton [10], the difficulties arising from a lack of documentation and a fixed address, together with the fragmentation of services, reinforce the health exclusion of this population [24] and perpetuate their invisibility in official statistics and in the design of public policies tailored to their needs. In line with Pleace [18], bureaucracy prevents many women from accessing regular medical care, which contributes to the worsening of physical and mental health problems and generates stress, anxiety and frustration that hinder their recovery and perpetuate their homelessness.

Another relevant finding is the impact of stigma and discrimination on the relationship between women experiencing homelessness and healthcare personnel. This stigma not only generates mistrust, but also affects their mental health, adherence to treatment and willingness to seek help in the future [38]. Previous studies have already documented the mistrust these women face in health services, as well as their perception of being treated with disdain or a punitive approach [28,29]. In this study, professionals confirmed that many women avoid going to health centers due to previous experiences of dehumanizing treatment and gender stereotypes that influence the care they receive. This finding is consistent with the literature that emphasizes the need for training in gender awareness and perspective among healthcare teams [19], as well as in understanding trauma and caring for vulnerable populations. Thus, in line with the findings of Alonso Pardo et al. [16], it is essential to create gender-specific social resources to prevent sexual assault and harassment, thereby ensuring safe and supportive spaces for women experiencing homelessness.

The lack of a comprehensive approach and gender perspective in healthcare also emerges as a central issue. Despite the fact that gender-based violence is a recurring experience in the lives of women experiencing homelessness [3,19], affecting their physical and mental health and increasing their risk of developing disorders such as depression, anxiety and post-traumatic stress [32], the professionals interviewed agreed that the healthcare system is not equipped to detect or address this issue effectively. As Matulič Domandzič et al. [21] point out, the invisibility of these experiences within social protection systems perpetuates their exclusion and hinders the implementation of effective intervention strategies. The absence of specific protocols and the lack of coordination between health and social services limit the response capacity in situations of violence and revictimize those affected, generating feelings of guilt, shame and hopelessness, and hindering their ability to overcome trauma and rebuild their lives.

Another significant finding is the scarcity of specialized resources for women experiencing homelessness with mental health and addiction problems. This scarcity hinders the implementation of a comprehensive approach that addresses the multiple needs of these women, including medical care, psychological support, and social counselling. The literature has identified mental health as one of the most affected dimensions in this population [32]. However, the present study highlights the absence of adequate treatment mechanisms, which leads to medicalization without therapeutic follow-up [25]. Long waiting lists and lack of access to comprehensive treatment hinder recovery and perpetuate the psychological and psychiatric problems of these women [33].

Bureaucracy and lack of coordination between health and social services were other barriers highlighted. The fragmentation of protection systems prevents a coordinated response to the needs of women experiencing homelessness, such as when a woman is referred from a health center to a social service without being adequately informed about the procedures she must follow, resulting in discontinuous and disjointed processes that hinder continuity of care [1,27]. This situation is exacerbated by the lack of documentation and a permanent address, which, as Botija et al. [24] point out, reinforce the health exclusion of this population. As suggested by Matulič et al. [21], it is essential to strengthen inter-institutional coordination mechanisms to ensure a comprehensive approach, such as the creation of multidisciplinary teams that work in a coordinated manner, the establishment of clear and simple referral protocols, and the implementation of shared information systems. In line with the proposals of Alcántara and Arredondo [9], it is necessary to implement a person-centered care approach that considers the particularities of female homelessness and the experiences of violence that often accompany it.

Finally, the lack of prevention and health education strategies specifically targeting this population is identified. This shortcoming, in line with the introduction on the invisibility of female homelessness [4,6,7], perpetuates health inequalities and limits the exercise of their fundamental rights. The absence of preventive programs, coupled with women’s lack of awareness of their health rights, perpetuates their exclusion from the health system and exacerbates their living conditions. As indicated in previous studies [34], homelessness not only increases the burden of disease but also reduces life expectancy, emphasizing the urgency of tailored health interventions.

## 5. Conclusions

The issue of female homelessness and the barriers these women face when accessing healthcare requires urgent attention in the design of social and health policies. These policies must be comprehensive, intersectional, gender-sensitive and multidimensional. Structural obstacles, such as lack of documentation, administrative fragmentation, and the absence of a gender perspective in healthcare, combine with stigma and discrimination to perpetuate their exclusion from the health system and deepen their vulnerability. As the participating professionals noted, the lack of a permanent address, bureaucratic hurdles and persistent stigma limit access to preventive care, mental health services and gynecological care.

Addressing these barriers requires coordinated, sustained and structural action. First, awareness and training programs for healthcare personnel must be strengthened to address stigma, discrimination and gender-based violence, promoting dignified, respectful and culturally competent care. These programs should improve communication with patients, recognize diverse trajectories and incorporate trauma-informed and gender-sensitive approaches. Second, simplifying administrative procedures, through one-stop service points, case management and flexible documentation requirements, would mitigate the structural exclusion faced by many homeless women. Third, investment in specialized services is needed, particularly in mental health and addiction care, with multidisciplinary teams capable of offering outpatient care, crisis intervention, hospitalization and long-term follow-up adapted to their needs.

Inter-institutional coordination also plays a central role in ensuring comprehensive and continuous care. Strengthening communication between health, social and housing services through intersectoral committees, shared protocols and integrated information systems would improve continuity and prevent women from being lost in bureaucratic processes. Finally, involving women experiencing homelessness in the design, implementation and evaluation of policies and programs is essential. Advisory councils, participatory processes and mechanisms to incorporate their perspectives would contribute to interventions better aligned with their realities.

Regarding methodological considerations, it is important to clarify that characteristics such as a qualitative design and purposive sampling are not limitations but inherent features of naturalistic inquiry, aligned with the epistemological foundations of this study. As Lincoln and Guba [46] emphasize, qualitative research prioritizes contextual meaning, credibility and depth over representativeness and statistical generalization. The focus on professionals’ perspectives is therefore appropriate to the exploratory aim of understanding institutional and structural barriers affecting homeless women.

Nonetheless, the study acknowledges methodological considerations relevant to qualitative inquiry. Consistent with Malterud’s [47] reflections, the researchers’ prior experiences and interpretative lenses may have influenced the analytical process, and although reflexive discussions and triangulated coding were implemented, deeper reflexivity could have further strengthened the credibility and confirmability of the findings. In addition, the absence of participant validation (member checking) may have limited the opportunity to contrast interpretations with the professionals involved in the focus groups. Moreover, it would have been valuable to ask participating professionals for suggestions on how to include women experiencing homelessness themselves in separate individual interviews, as such insights could have provided practical guidance for designing future studies that directly incorporate their voices. These considerations outline the interpretative boundaries of the study while reinforcing its methodological coherence.

Beyond these issues, several further limitations should be acknowledged. Although the qualitative design and purposive sampling are suitable for the exploratory nature of the study, the sample size and the exclusive inclusion of social intervention professionals limit the diversity of perspectives captured. The exclusive reliance on focus groups… may also have constrained the expression of more sensitive or divergent viewpoints. Furthermore, the study was conducted in three cities within a single national context, so contextual variations may limit transferability to other regions or countries. These limitations do not undermine the value of the study but highlight opportunities to expand and strengthen future research in this field.

Building on these limitations, future research should incorporate the direct perspectives of women experiencing homelessness themselves, both through individual interviews and women-only discussion spaces, and consider mixed-methods and longitudinal designs to examine the evolution of health needs, access trajectories and intervention outcomes over time. Expanding reflexive practices and incorporating participant validation processes would further enhance the credibility and applicability of findings. Despite these considerations, the present study offers a robust foundation for action and underscores the urgent need to prioritize the health of women experiencing homelessness on political and social agendas, ensuring access to dignified, high-quality and gender-sensitive healthcare tailored to their specific needs.

## Data Availability

The original contributions presented in this study are included in the article. Further inquiries can be directed to the corresponding author.
